# Preventing and developmental factors of sustainability in healthcare organisations from the perspective of decision makers: an exploratory factor analysis

**DOI:** 10.1186/s12913-023-09689-w

**Published:** 2023-07-25

**Authors:** Mario Calabrese, Shefqet Suparaku, Savino Santovito, Xhimi Hysa

**Affiliations:** 1 University La Sapienza, Rome, Italy; 2 Polis University, Tirana, Albania; 3 University of Bari, Bari, Italy

**Keywords:** Healthcare system, Healthcare sector, Healthcare organisations, Sustainability, Sustainable development, Decision makers, Exploratory factor analysis

## Abstract

**Background:**

UN Sustainable Development Goals are part of the political agenda of most developed countries. Being a developing country, Albania has only recently adhered to this trend. Prior research at national level has sporadically focused on environmental sustainability, neglecting a holistic view of the phenomenon. To fill this gap, this study aims to explore preventing and developmental factors of sustainability in healthcare organisations from the perspective of decision makers by relying to a Triple Bottom Line approach.

**Methods:**

Data were collected through a questionnaire administered to healthcare facilities and analysed through the Exploratory Factor Analysis. Findings revealed that the factors influencing the sustainability of the national healthcare system were five: Barriers of Organisational Sustainability; Stakeholders Pressure (regarding sustainable issues); Awareness (knowledge and measures taken for sustainability); Institutional Engagement; and Personal Interest and Involvement. The underlying factors included 19 items suitable for this sample, representing 64.371% of the total variance.

**Results:**

The findings show the existence of 4 factors: Barriers of Organisational Sustainability, Stakeholders Pressure regarding Sustainable issues, Awareness/knowledge and measures taken for sustainability, Personal Interest and Involvement.

**Conclusions:**

It is evident that national health organisations should continuously improve its strategies to be consistent with the sustainable development goals of international organisations, so that their initiatives could reflect the integration of sustainability approaches at the organisational level.

## Introduction

Despite the continuous effort of several countries to experiment with innovative methods of organizing and supporting better health care delivery to encounter the increasingly disparate needs of patients, it has been difficult to translate the necessary changes into sustainable and effective strategies on a large scale [1]. The COVID-19 pandemic has caused enormous challenges to health systems around the world (Rucker et al., 2021), especially for traditional health systems which have demonstrated inherent problems in preparing and preventing the current situation, with consequences in economic and social terms [2] This is a reminder of a radical rethinking of the way to solve future problems to avoid being caught unprepared for new epidemics. Furthermore, it is also an appeal to all actors involved in the decision-making and governance process to face complex problems with flexible solutions in challenging contexts so that everyone can continue to make progress in achieving the SDGs.

The complexity of the challenges launched by the 2030 Agenda require an integrated vision of the different dimensions of sustainable development (economic, environmental, and social) that are closely related and balanced to each other [3], pursued on the basis of a careful evaluation of each other’s interrelationships [4]. A comprehensive and correct reading of the three components, which considers exactly how they are evolving [5] will allow the achievement of sustainable development and sustainability in different sectors.

With regards to the healthcare sector, the need to increase efficiency and improve the quality of service in recent years has led to an exponential growth of interest in the application of sustainable principles to healthcare [6, 7], while increasing awareness and improvement of responsible behaviour in the “proper” management of internal resources [8]. The need to root health policy and planning within environmental and social approaches is part of a wider concern of the health care organisations for organisational survival, continued development, and improvement of health services [9, 6]. However, as some authors argue [10, 11], there is no empirical analysis that relates the “sustainable development” of health policies and programs.

Assuming that the SDGs objectives are conceived as universal, and taking into consideration that all countries are called to contribute to the definition of their own sustainable development strategy that agrees with international principles [3], a question arises: what are the main factors influencing sustainability in the health sector and how is Albania acting towards achieving sustainable development goals?

The growing awareness ensuring rapid and effective solutions in terms of health and well-being for its population, has required in the recent years the need to restructure the National Health System by referring to universal public health systems and standards. This has been subject to numerous revisions, modifying the functioning of Albanian healthcare. The recent opening of EU integration negotiations has influenced the commitment of Albanian Government towards the achievement of SDGs of the 2030 Agenda. However, the initiatives and actions aimed at achieving the set of objectives are still in an embryonic stage, comparing to some Balkan countries.

The action to be taken is a challenging and complex task [12], and a public health system is based on choice, and it is as sustainable as public opinion and politicians think it should and can be [13]. Consequently, it is up to political, economic, and organisational decision-makers to structure flexible strategies in the face of rapid and continuous changes that modify the needs and expectations of citizens, reallocating available resources [14]. The responsibility of satisfying the needs of citizens and solving relational problems affect the survival of the system in a given context, developing conditions of consonance (in terms of the ability to relate to the outside world) and resonance (as an interactive system able to generate harmony between the parts) with the other entities involved in the basic dynamics of the system [15–17].

Embedded in broader social, industrial, and political systems, health care organisations tend to be seen as the complex systems that influence pace and dissemination on sustainable practices and policies, even though they sometimes act in ways that are not always linear and predictable [18]. Although the competitive advantage that integration of sustainable principles in core policies and overall performance goals can bring, [19] states that there is no “one size fits all” sustainable approach and the challenge for organisations about sustainability lies in determining what their organisational strategy should be. The lack of a specific strategy could be searched in the habits of the organisations to calculate everything in financial terms and in the difficulty of aligning the other non-financial aspects, indispensable for sustainability [20], or in the absence of a “subjective interpretation” of the governing body during the planning of organisational sustainability in the specific context [21], as well as in the absence of a shared strategy and interpretative keys on how the development objectives have to be pursued [22].

Through the proposed definition of *Sustainable Development* [23], which includes three pillars of sustainability: environmental, social, and economic, and in support of the *Triple Bottom Line* concept introduced by [24], this study aims to explore the adoption of sustainable development approaches in the Albanian Healthcare System, focusing more specifically on the hospitals of the national system, and how this concept is interpreted by decision makers. Given the importance of the general topic, the lack of research on this sector and the importance of these institutions, the study seeks to:

- assess the level of adherence of sustainable principles within healthcare organisational structures, and verify whether the assessment expressed by decision-makers is in line with the initiatives and practices of international principles;
- understand which are the most important factors and the interested parties (*stakeholders*) that push decision-makers to inhibit sustainability in the operational reality;
- identify the most significant barriers that prevent the implementation of sustainable programs and practices in national health structures.

## Literature review

Although the concept of sustainability in recent years can no longer be limited only in manufacturing organisations but finds application in different service sectors [25], the research on its significance in the health sector is still not comprehensive [26]. Due to the difficulty of defining it, measuring, and making it operational, only a few organisations in this sector have tried to put this approach into practice [27]. For some health systems that find it difficult to meet the growing health demands of citizens due to limited financial resources available [28], the strengthening of sustainability becomes a key concept and a principle that guides the health sector to development and getting through successfully [29]. According to [30], health sustainability refers precisely to the ability of systems to promote the long-term health and well-being of people within society. Furthermore, [31] consider as sustainable those health systems that manage to balance the interests of stakeholders, while having the ability to improve, innovate and develop continuously. The sustainability of a system in this perspective involves a balance between cultural, social, economic, and environmental factors [32].

As for the social approach, the sustainability of health systems must be sought on the ability of organisations to redesign relationships with patients, considering legitimacy, trust, and social value [33]. The relationship that is established tends to emerge as a means of creating and maintaining value within organisations that aims to provide superior quality care to citizens and patients [34]. Other authors [35] pay attention to human resources, as they argue that sustainability in healthcare organisations is one of the most important aspects to be considered by healthcare professionals, as individuals who can promote sustainable behaviours [36]. For example, investments in lifelong learning and adequate education to support the skills and quality of human resources [37], together with the recruitment of “green staff” and the need for ecological orientation towards green issues [38], as well as the creation of a healthy work environment that increases the job satisfaction of its staff [35], are just some key factors in the strategic management of human resources, which contribute not only to improving the organisation’s performance and results [39] but also in strengthening sustainable health systems. On the other hand, healthcare managers must prepare initiatives for the distribution and conservation of human resources, putting workers first and at the same time building cooperation between operators of the sector and the government [40].

While trying to best meet patients’ needs, healthcare organisations consume vast amounts of resources, with a direct impact on climate change and human health [41]. Faced with this situation, it has become a necessity to find a balance between environmental and economic concerns, in the combination of the three key factors: quality of assistance provided, responsible fiscal financing, minimum environmental impact [42]. In other words, organisations must have the financial resources to create friendly environmental initiatives such as recycling, energy efficiency, water conservation etc. [43]. Current initiatives work closely with other initiatives that reduce explicit environmental risks such as: supply chain management activities [44] green procurement initiatives and nature-based solutions [45, 46], the trend to collaborate with suppliers who pay attention to environmental issues [47], compliance with the legislation and guidelines required in the field of environment and safety for sustainable procurement [48], extension of construction techniques to create a healing environment [49] or even the construction of the *Green Hospital*, as an answer to all these issues, while improving conditions for employees and patients [50]. However, the lack of financial resources is seen as one of the most critical internal challenges for most healthcare sectors, preventing organisations from implementing sustainable practices [51].

The continuous increase in healthcare expenses and the overall weight that the sector absorbs in the national budget [52], together with the unavailability resources to effectively meet the care needs of users [53], as well as the indifference of governments to allocate sufficient resources to cover the system’s obligations, in terms of costs and continuous improvement of quality and financial performance [54], are just some of the critical factors that threaten economic sustainability in the healthcare sector. Indeed, if on one side sustainability in healthcare operations is achieved when a quality service is provided, trying to balance the resources and patients’ needs [55], and the implementation of sustainability would result in both a financial and qualitative improvement for health care [56], on the other hand, political decisions are those that impose fiscal constraints in a country and determine the size, budget allocations and priorities of the national system [57].

Other factors can favour the adoption of sustainable initiatives in healthcare facilities, such as: the advanced use of technology and the development of e-health information strategies [28], the pressure exerted by society on the use of resources [58], as well as the pressure exerted by the stakeholders on the provision of the service to the patients [59], codes of conduct and codes of ethics [60], patient empowerment and value co-creation [61]. Recent studies indicates, in fact, the relevant role of technology- whose impact is unavoidable also for healthcare [62]- for achieving sustainability, for example with regards to healthcare supply chains, of deep learning and artificial intelligence (AI) [63], virtual support systems [64], digital transformation [65] as well as the role of blockchain technology, which can help-according to [66]- circular economy as well as reducing carbon footprint in healthcare activities. Moreover, the digitalization of healthcare is also defined Healthcare 4.0, which includes the use of technologies such as m-health, wifi health, e-health [67].

Other authors [68], while trying to measure sustainability in the healthcare sector, identify as main factors: lean management, patient and employee satisfaction, continuous improvement, Corporate Social Responsibility (CSR), brand and accreditation. Furthermore, the perception and attitude of managers towards sustainability are also factors that need to be taken into consideration, as determining elements that influence organisational sustainability strategies [69]. To this end, [70] have underlined how management is essential in the healthcare sector, whose performances are strictly connected to managerial approaches, especially practices, leadership, manager characteristics and cultural attributes.

[71], moreover, explored the role of green human resources on sustainable performances in the healthcare sector. In particular, through a survey, they revealed that the most influential practices were green hiring (which environmental costs are lower than formal training courses) and green training and involvement.

On the other hand, other studies in this field revealed both internal and external barriers, which slow down the will and capacity of a health organisation to pursue sustainability initiatives: barriers of environmental approach [72], barriers on social approach [73, 74]; barriers on economic approach.

## Methodology

To investigate organisational sustainability in the national healthcare context in a more detailed way, we decided to explore this topic by building a semi-structured questionnaire as a survey tool to be administered through virtual channels. The questions were developed from the previous literature review. The questionnaire was divided into 3 parts: Part 1, named as “*Personal Awareness and Interest on Sustainability*”, which intended to assess the level of knowledge and the degree of personal interest in sustainability issues of all participants for the sustainability issues; Part 2, named as “*Organisational Sustainability*”, focused on the analysis of the effect that the individual components of sustainability have on healthcare facilities, interpreted from the perspective of decision makers; Part 3, named as “*Demographic Characteristics*”, included general characteristics, gender, age, education, work experience and workplace. The questionnaire was tested for reliability using 19 variables, with Cronbach’s alpha coefficient of 0.636, making it a very reliable tool.

Thestatistical sample refers to the entire hospitals that provide health services in the national territory, excluding from the analysis other small structuressuch as clinics or residential centres. The survey was administered by the Data Centrum Research Institute (https://www.datacentrum.al/en/), that is equipped with the experience and its own databases from the Ministry of Health. Consequently, making the data collection process more reliable. The questionnaire was addressed to employees of healthcare organisations who play a key role in the management and supervision of health facilities (such as hospital general managers, health directors, deputy directors, technical directors, administrators of integrated management poles, heads of financial offices, HR directors, etc.).

544 people were contacted by telephone, of which only half responded mainly to the call, expressing their willingness to be part of the research. Participants were given the necessary link to access the Survey Monkey platform to fill out the questionnaire. 120 questionnaires were collected, corresponding to a response rate of 17.6%, but 31 of them were removed as incomplete and with strong internal inconsistencies. In the end, a total of 89 complete questionnaires were obtained, with a final response rate of 13%. Once the questionnaires were collected, the quality assurance phase was carried out to provide a clean database. During this phase, some of the results were manually coded and subsequently grouped and inserted in a final database. Study participants were assured of their anonymity and confidentiality.

Given the exploratory-descriptive nature of the survey, the first phase of our research provides a general overview of the characteristics of our sample; then, the Exploratory Factor Analysis (EFA) was applied to determine the underlying structural dimension of the variables identified from the literature review. Through this method, it was useful to identify the main contributing factors to the implementation of sustainable practices at an organisational level. Varimax Rotation was applied to determine the dimensionality of the factors. Each of the identified factors fulfilled the satisfactory level of internal consistency and acceptability. The items in turn were measured using a 5-point Likert scale in which 1 represents “the lowest level” of the evaluation and 5 represents “the highest level”.

## Findings

Among 89 respondents (i.e., healthcare professionals), 47 (53%) were female and 42 (47%) were male. Based on their position within the organisation, 17% of them were hospital managers, 8% were deputy directors (technical directors), 16% were medical managers, 24% were department heads, and 36% in other decision-making positions. As for the distribution of participants according to the organisation they belong to, it is noted that 78% are part of the public structures, compared to 12% of the private ones. Such a high percentage of participants from the public sector is also the reason for the dominance of public hospitals in offering health care in the national territory. The age of most participants ranged from 35 to 54 years, with a mean age of 46.1 years. Most of the participants (79%) had a medical degree.

To extract the number of principal factors, an exploratory factor analysis (EFA) was performed, using the principal components method, on the 19 variables measured with the Likert scale. At the end of this analysis, and through the Varimax Rotation Method, 5 main factors that influence health sustainability in national hospitals were identified. The analysis was performed only once, and the 5 factors represent 64.371% of the total variance. The suitability analysed by Kaiser – Meyer – Olkin (KMO) is 0.776, which means it is very suitable for this analysis, and Bartlett’s sphericity test was found to be significant (Table 1).

**Table 1:** Summary statistics

**Cronbach’s Alpha**		0.636
**Kaiser-Meyer-Olkin Measure of Sampling Adequacy**		0.776
**Bartlett’s Test of Sphericity**	Approx. Chi-Square	637.195
	Df.	171
	Sig.	0.000

N = 89; Number of Items = 19

For the five factors the eigenvalues, percentage of variance and accumulative percentage of variance were observed (Table 2). The first factor, which includes 6 items, represented 25.703% of the variance. The second factor, which includes 3 items, represents 15.512% of the variance. The third, fourth and fifth factors, which include 3, 4, 3 items each of them, represent 8.84%, 7.807% and 6.509% of the variance, respectively, as shown in Table 2. As also mentioned above, the 5 factors formed account for 64.371% of the total variance and this is a moderately high value.

**Table 2:** Number of Factors Related to Eigen Value and Explanatory Percentage of Variance

Factors	Initial Eigenvalues			Rotation Sums of Squared Loadings		
	Total	% of Variance	Cumulative %	Total	% of Variance	Cumulative %
**1**	4.883	25.703	25.703	3.327	17.511	17.511
**2**	2.947	15.512	41.215	2.485	13.082	30.592
**3**	1.680	8.840	50.055	2.373	12.490	43.082
**4**	1.483	7.807	57.862	2.278	11.987	55.069
**5**	1.237	6.509	**64.371**	1.767	9.302	**64.371**

After this phase, we selected the factors with the classical method of Orthogonal Rotation. The following table (Table 3) shows the loading of each item on the 5 extracted factors. Items with factorial loads less than 50 have been removed from the model. The first factor that emerged contains 6 items and is called *Barriers of Organisational Sustainability (BOS)*. Factor loads vary with a maximum of 0.818 on the item *ESB (External Social Barriers)* and with a minimum of 0.544 on the item *EEcB (“External EconomicBarriers”)*. Each of these items refers to internal and external barriers that prevent the implementation of programs and initiatives towards achieving sustainable goals by reconciling the social, economic, and environmental dimensions.

**Table 3:** Rotated Component Matrix

Named Factors	Components	Factor loading				
		1	2	3	4	5
**Barriers of Organisational Sustainability (BOS)**	External Social Barriers (ESB)	0.818				
	External Environmental Barriers (EEB)	0.766				
	Internal Social Barriers (ISB)	0.749				
	Internal Economic Barriers (IEcB)	0.691				
	Internal Environmental Barriers (IEB)	0.683				
	External Economic Barriers (EEcB)	0.544				
**Stakeholders Pressure regarding Sustainable issues (SPSs)**	Stakeholders positive Pressure regarding Social issues (SPS)		0.868			
	Stakeholders positive Pressure regarding Economic issues (SPEc)		0.793			
	Stakeholders positive Pressure regarding Environmental issues(SPE)		0.772			
**Awareness/knowledge and measures taken for sustainability (AKMS)**	Level of awareness/Knowledge on Sustainability main components (LKS)			0.807		
	Level of implementation of the Sustainability components (LIS)			0.789		
	Guidelines and Training materials on Sustainability components(GTS)			0.753		
**Institutional Engagement (IE)**	Institutional Environmental Engagement (IEE)				0.840	
	Institutional Approach to Environmental issues (IAE)				0.727	
	Institutional Social Engagement (ISE)				0.667	
	Institutional Economic Engagement(IEcE)				0.557	
**Personal Interest and Involvement (PII)**	Level of Interest on environmental, economic, and social Sustainability(LItS)					0.745
	Personal Importance of the environment/Social issues (PIS)					0.737
	Level of Personal Involvement (LPI)					0.569

The second factor, “*Stakeholders Pressure regarding Sustainable issues”(SPSs*), includes 3 items, with loads of factors ranging from a maximum of 0.868 on the item SPS (Stakeholders positive Pressure regarding Social issues) and a minimum of 0.772 on the item SPE (Stakeholders positive Pressure regarding Environmental issues). These items refer to the main stakeholders that push the healthcare organisation to engage in sustainable development issues, such as patients, employees, local communities, suppliers, media etc.

The third factor, called “*Awareness/knowledge and measures taken for sustainability”(AKMS)*, refers to the knowledge of decision makers on organisational sustainability and what are the measures taken to implement sustainable principles. This factor includes 3 items, with loads of factors ranging from a maximum of 0.807 on the item LKS (Level of awareness/knowledge on Sustainability main components) and with a minimum of 0.753 on the item GTS (Guidelines and Training materials on Sustainability components).The fourth factor, “*Institutional Engagement”(IE)*, includes 3 items, in which factor loads vary with a maximum of .840 on the IEE item (Institutional Environmental Engagement) and with a minimum of .557 on the IEcE item (Institutional Economic Engagement). Each of these items refers to the main reasons that commit the organisation to sustainable initiatives.

The last factor, “*Personal Interest and Involvement” (PII*), refers to the awareness and personal interest of decision-makers for sustainability. This factor includes 3 items, with factor loads that vary with a maximum of 0.745 on the LItS item (Level of Interest on environmental, economic, and social Sustainability”) and with a minimum of 0.569 on the LPI (Level of Personal Involvement) as shown in Table 3.

## Discussion

This study aimed to explore preventing anddevelopmental factors of sustainability in healthcare organisations from the perspective of decision makers, while offering a more holistic framework within which the most salient factors and the most significant obstacles are summarized in the implementation of sustainable strategies. The results of our study suggested five main factors that influence the most the sustainability issues of the country’s healthcare facilities:

- *Personal interest and involvement*: our results showed that most of the interviewees show a great interest in the issues of sustainability and sustainable development, as well as feel the awareness of being involved in helping to bring change in this direction. Unlike the other approaches, the social dimension carries a greater interest among respondents.
- *Awareness and measures taken for sustainability*: even if the amount of general knowledge was high, there is a lack of understanding of the individual components when it comes to organisational sustainability, also confirming the fact that almost half of the interviewees admit that “sustainability policies are a new concept for one’s own organisation”, and are not yet integrated into the strategic organisational policies, which should be in line with the Sustainable Development Goals established by the UN. However, the private sector prevails both in terms of measures taken and in the degree of involvement on sustainability practices with respect to the public.
- *Barriers of organisational sustainability*: regardless of whether they are internal or external barriers, the results showed that decision makers attribute greater value to financial costs as the main factor preventing the implementation of sustainable programs and initiatives. Furthermore, comparing the results of the 3 dimensions of sustainable development approaches, the external barriers are those that are perceived as more relevant than the internal ones, probably due to the tendency for individual participants to delegate issues outside the institution to which they belong.
- *Stakeholders pressure*:Regarding the pressure exerted on the issues of organisational sustainability, the results obtained allow us to state that decision makers generally perceive a very low level of pressure from the various stakeholders, assuming in this case a degree of little importance. This is in negative correlation with the development of proactive strategies, as the lower the perceived pressure, the less the organisation feels the responsibility to implement initiatives that go far beyond what the law states, and what stakeholders expect.
- *Institutional engagement*: Regarding the factors that drive the healthcare organisation to engage in sustainable issues, most respondents say on an overall level that implementing the appropriate initiatives brings added value to the organisations and is the right thing to do, as it improves the image and performance, as well as increases the well-being of employees.

Concluding the analysis, it is possible to say that national health organisations should continuously improve its strategies to be in line with the sustainable development goals of international organisations, so that daily initiatives reflect the integration of sustainability approaches at the organisational level.

Despite of its potential, this study comes with few limitations that must be addressed in future research. A first limitation is related with the data collection process itself that has been affected by the pandemic. The measures adopted for the prevention of Covid-19 during the period of this research have created difficulties in accessing health institutions, leading to the limits of conducting the survey in other ways and with a greater number of participants, despite our will to involve a larger sample and extend the study to all hospitals throughout the country. Another limitation is about the subjective bias related to perceptual processes of participants. Thus, studies involving subjective perception are difficult as each respondent can feel it differently, altering the final dataset.

With regards to potential implications, despite of the limitations expressed above, we believe that the contribution of this study is very important and unique in the national context, capable of producing a stable and replicable research structure in the future. On one hand, it can have practical implications as it can help professionals in evaluating and choosing the most relevant factors to improve the sustainability of their organisations. On the other hand, it is a good starting point for further research on organisational sustainability in the healthcare sector. The use of more in-depth and sophisticated qualitative and quantitative analysis can be valid tools for evaluating the causal relationships of sustainability factors in healthcare organisations, offering a consistent model that prioritizes sustainable strategies in specific contexts. Furthermore, a comparison between public and private hospitals in the context of measuring sustainability could be an interesting direction of future research.

## Data Availability

The datasets used and/or analysed during the current study available from the corresponding author on reasonable request.

## References

[1] Nolte E, Kluge H, Figueras J (2018). How do we ensure that innovation in health service delivery and organisation is implemented, sustained and spread?. Health Systems for Prosperity and Solidarity.

[2] Sun S, Xie Z, Yu K (2021). COVID-19 and healthcare system in China: challenges and progression for a sustainable future. Global Health.

[3] UN (2015). Transforming our world: the 2030 agenda for Sustainable Development.

[4] Scharlemann JPW, Brock RC, Balfour N (2020). Towards understanding interactions between Sustainable Development Goals: the role of environment–human linkages. Sustain Sci.

[5] Nilsson M, Griggs D, Visbeck M (2016). Map the interactions between sustainable development goals. Nature.

[6] Hudson CG, Vissing YM (2013). Sustainability at the edge of chaos: its limits and possibilities in public health. Biomed Res Int.

[7] Gambarov V, Sarno D, Hysa X, Calabrese M, Bilotta A (2017). The role of loyalty programs in healthcare service ecosystems. TQM J.

[8] da Filicaia MG. Le molte facce della sostenibilità. Come mantenere universale ed equo il servizio sanitario. Forward, Sostenibilità, vol. 1, 13, Maggio 2019. Available at https://forward.recentiprogressi.it.

[9] Stokols D (1996). Translating social ecological theory into guidelines for community health promotion. Am J Health Promotion.

[10] Njiro E (2002). Introduction. Sustainable development: an oxymoron?. Esther Agenda.

[11] Gruen R, Elliott J, Nolan M, Lawton P, Parkhill A, McLaren C (2008). Sustainability science: an integrated approach for health-programme planning. Lancet.

[12] Ciko I. Albania, report on the harmonisation of sustainable development goals with existing sectoral policies. United Nations (UN), January 30; 2018.

[13] Health Council of Canada (2008). Sustainability in Public Health Care: what does it Mean? A panel discussion report.

[14] Fineberg HV (2012). *A* successful and sustainable Health System — How to get there from Here. N Engl J Med.

[15] Barile S (2009). Management sistemico vitale(Vol. 1).

[16] Golinelli GM (2010). Viable Systems Approach (VSA). Governing Business Dynamics.

[17] Barile S, Saviano M, Iandolo F, Calabrese M (2014). The viable systems approach and its contribution to the analysis of sustainable business behaviors. Syst Res Behav Sci.

[18] Plsek PE, Greenhalgh T (2001). Complexity science: the challenge of complexity in health care. BMJ (Clinical research ed).

[19] Wales T. Organisational sustainability: what is it, and why does it matter? Rev Enterp Manag Stud. 2013;1(1):38–49.

[20] Kielstra P. Doing good: business and the sustainability challenge (online). Economist Intelligence Unit Report; 2008.

[21] Hysa X, Calabrese M, Bilotta A, Salaj F (2016). A service–system paradigm for governing corporate sustainability: the (forgotten) role of governing body in shaping sustainability and context. Int J Environ Health.

[22] GCAP Italia (a cura di). Sviluppo sostenibile: per chi? Una visione critica per la coerenza delle politiche italiane ed europee. Rapporto di monitoraggio sull’applicazione dell’Agenda 2030 in Italia; 2018. p. 2.

[23] Brundtland Commission. Report of the World Commission on Environment and Development. Our Common Future; 1987.

[24] Elkington J. Cannibals with forks: the triple bottom line of 21st century business. Capstone: Oxford; 1997.

[25] Stylos N, Vassiliadis C (2015). Differences in sustainable management between four-and five-star hotels regarding the perceptions of three-pillar sustainability. J Hosp Market Management.

[26] Hussain M, Malik M, Neyadi A (2016). AHP framework to assist lean deployment in Abu Dhabi public healthcare delivery system. Bus Process Manage J.

[27] Machado CML, Scavarda A, Kipper LM, Santa R, Ferrer MA (2015). Sustainability at the healthcare organisations: an analysis of the impact on the environment, society, and economy. Chem Eng Trans.

[28] Coiera E, Hovenga EJS (2007). Building a sustainable health system. IMIA Yearbook of Medical Informatics.

[29] Romanelli M. Towards sustainable health care organisations. Manag Dyn Knowl Econ. 2017;5(3):377–94.

[30] Aquino RP, Barile S, Grasso A, Saviano M. Envisioning smart and sustainable healthcare: 3D Printing technologies for personalized medication. Futures. 2018;103:35–50.

[31] Lifvergren S, Huzzard T, Docherty P. A Development Coalition for sustainability in Health Care. In: Docherty P, Kira M, Shani ABR, editors. Creating sustainable Work Systems. Developing Social Sustainability Routledge; 2009.

[32] Prada G, Grimes K, Sklokin I. Defining health and health care sustainability. Ottawa: The Conference Board of Canada; 2014.

[33] Gilson L (2003). Trust and the development of health care as a social institution. Social Sci &Medicen.

[34] Straten GF, Friele RD, Groenewegen PP (2002). Public trust in dutch health care. Soc Sci Med.

[35] Marimuthu M, Paulose H (2016). Emergence of sustainability based approaches in Healthcare: Expanding Research and Practices. Procedia-Social and Behavioral Sciences.

[36] Colombo G, Gazzola P (2014). Aesthetics and Ethics of the sustainable organisations. Eur Sci J ESJ.

[37] Kamoche K (1996). Strategic Human Resource Management within a resource-capability view of the firm. J Manage Stud.

[38] Alipour N, Sangari SM, Nazari-Shirkouhi S. Investigating green human resource practices in the healthcare sector: a joint application of balanced scorecard and SIR method. In: 15th Iran international industrial engineering conference (IIIEC), Yazd, Iran. 2019. p. 283–8.

[39] Stanton P, Leggat SG (2007). Lost in translation: exploring the link between HRM and performance in healthcare. Hum Resource Manage J.

[40] Chen L, Evans T, Anand S, Bufford H, Chawdhury M, Cueta M, Dare L, Dussault G, Elzinga G, Fee E, Habte D, Hanvoravanghcai P, Jacobs M, Kurowski C, Michael S, Solimano G (2004). Human resources for health: overcoming the crisis. The Lancet.

[41] Dhillon VS, Kaur D. Green hospital and climate change: their interrelationship and the way forward. J Clin Diag Res. 2015;9(12):LE01–5. 10.7860/JCDR/2015/13693.6942 PMC4717717 26814377

[42] Jameton A, McGuire C (2002). Toward sustainable health-care services: principles, challenges, and a process. Int J Sustain High Educ.

[43] McGain F, Naylor C (2014). Environmental sustainability in hospitals - a systematic review and research agenda. J Health Serv Res Policy.

[44] Carter CR, Carter JR (1998). Interorganisational determinants of environmental purchasing: initial evidence from the consumer products industries. Decis Sci.

[45] Rathi M, Wilburn S, Welter V, Dora C. UN Initiative on Greening procurement in the Health Sector from Products to Services, Landscape Analysis for the Technical Consultation. World Health Organisation (WHO); 2013. p. 1–63.

[46] Conti ME, Battaglia M, Calabrese M, Simone C (2021). Fostering sustainable cities through Resilience thinking: the role of Nature-Based solutions (NBSs): Lessons learned from two italian Case Studies. Sustainability.

[47] Tsioumpri K, Tsakni G, Goula A. Sustainable development in healthcare facilities. Case Study: Swedish and Greek Hospital. J Sustain Dev. 2020;13(4):178–90.

[48] Chiarini A, Opoku A, Vagnoni E. Public Healthcare practices and criteria for a sustainable procurement: a comparative study between UK and Italy. J Clean Prod. 2017;1–27.

[49] Kim S, Osmond P (2013). Analyzing green building rating tools for healthcare buildings from the building user’s perspective. Indoor and Built Environment.

[50] Teymourzadeh E, Yaghoubi R, Ghanizadeh G, Zaboli R (2022). Green Hospital; an overview of the most important indicators. Health Res J.

[51] Hoejmose SU, Adrien- Kirby AJ (2012). Socially and environmentally responsible procurement: a literature review and future research agenda of a managerial issue in the 21st century. J Purchasing Supply Manage.

[52] Palumbo R. Toward a new conceptualisation of health care services to inspire public health. Public national health service as a “common pool of resources”. Int Rev Public Nonprofit Mark. 2017;14(3).

[53] Porter M, Tiesberg E (2006). Re-defining health care delivery.

[54] Wildavsky A. Doing better and feeling worse: the political pathology of health policy. In: Peters B, editor. The art and craft of policy analysis. London: Palgrave Macmillan; 2018. p. 305–32.

[55] Faezipour M, Ferreira S (2013). A system dynamics perspective of patient satisfaction in healthcare. Procedia Comput Sci.

[56] Tudor TL (2007). Towards the development of a standardised measurement unit for healthcare waste generation. Resour Conserv Recycl.

[57] Coman A, Grigore A. Innovation as a driver of the sustainable healthcare systems: the case of Romania. J Innov Bus Best Pract. 2017.

[58] Paper R, Pinzone M, Lettieri E, Masella C (2012). Sustainability in Healthcare: combining Organisational and architectural levers. Regul Paper.

[59] Pereno A, Eriksson D (2020). A multi-stakeholder perspective on sustainable healthcare: from 2030 onwards. Futures.

[60] Rudnicka A. Codes of conduct and codes of ethics as tools used to support the idea of social responsibility in supply chains. Research Papers of Wrocław University of Economics, nr 464, ISSN 1899–3192; 2017.

[61] Polese F, Tartaglione AM, Cavacece Y. Patient empowerment for healthcare service quality improvements: a value co-creation view. In: 19th Toulon-Verona international conference, excellence in services. University of Huelva (Spain). 2016. p. 385–97.

[62] Garcia-Perez A, Cegarra-Navarro JG, Sallos MP, Martinez-Caro E, Chinnaswamy A (2023). Resilience in healthcare systems: Cyber security and digital transformation. Technovation.

[63] Azadi M, Yousefi S, Farzipoor Saen R, Shabanpour H, Jabeen F (2023). Forecasting sustainability of healthcare supply chains using deep learning and network data envelopment analysis. J Bus Res.

[64] Johnson K, Collins D, Wandersman A (2023). Developing a sustainability readiness strategy for health systems: Toolkit, interactive tools, and virtual support system. Eval Program Plan.

[65] Gomez-Trujillo AM, Gonzalez-Perez MA (2022). Digital transformation as a strategy to reach sustainability. Smart and Sustainable Built Environment.

[66] Upadhyay A, Mukhuty S, Kumar V, Kazancoglu Y (2021). Blockchain technology and the circular economy: implications for sustainability and social responsibility. J Clean Prod.

[67] Ahsan MM, Siddique Z (2022). Industry 4.0 in Healthcare: a systematic review. Int J Inform Manage Data Insights.

[68] Al Jaberi OA, Hussain M, Drake PR. A framework for measuring sustainability in healthcare systems. Int J Healthc Manag. 2017.

[69] Buysse K, Verbeke A (2003). Proactive environmental strategies: a Stakeholder Management Perspective. Strateg Manag J.

[70] Lega F, Prenestini A, Spurgeon P (2013). Is management essential to improving the performance and sustainability of Health Care Systems and Organizations? A systematic review and a Roadmap for Future Studies. Value in Health.

[71] Mousa SK, Othman M (2020). The impact of green human resource management practices on sustainable performance in healthcare organisations: a conceptual framework. J Clean Prod.

[72] Hillary R (2004). Environmental management systems and the smaller enterprise. J Clean Prod.

[73] Alotaibi A, Edum-Fotwe F, Price AD (2019). Critical barriers to social responsibility implementation within Mega-Construction Projects: the case of the Kingdom of Saudi Arabia. Sustainability.

[74] Zhang X, Shen L, Wu Y, Qi G. Barriers to implement green strategy in the process of developing real estate projects. Open Waste Manag J. 2011;4(1).

